# Automatic Modulation Classification for MASK, MPSK, and MQAM Signals Based on Hierarchical Self-Organizing Map

**DOI:** 10.3390/s22176449

**Published:** 2022-08-26

**Authors:** Zerun Li, Qinglin Wang, Yufei Zhu, Zuocheng Xing

**Affiliations:** College of Computer, National University of Defense Technology, Changsha 410073, China

**Keywords:** automatic modulation classification, hierarchical self-organizing map, high-order cumulants, amplitude moments, clustering models

## Abstract

Automatic modulation classification (AMC) plays a fundamental role in common communication systems. Existing clustering models typically handle fewer modulation types with lower classification accuracies and more computational resources. This paper proposes a hierarchical self-organizing map (SOM) based on a feature space composed of high-order cumulants (HOC) and amplitude moment features. This SOM with two stacked layers can identify intrinsic differences among samples in the feature space without the need to set thresholds. This model can roughly cluster the multiple amplitude-shift keying (MASK), multiple phase-shift keying (MPSK), and multiple quadrature amplitude keying (MQAM) samples in the root layer and then finely distinguish the samples with different orders in the leaf layers. We creatively implement a discrete transformation method based on modified activation functions. This method causes MQAM samples to cluster in the leaf layer with more distinct boundaries between clusters and higher classification accuracies. The simulation results demonstrate the superior performance of the proposed hierarchical SOM on AMC problems when compared with other clustering models. Our proposed method can manage more categories of modulation signals and obtain higher classification accuracies while using fewer computational resources.

## 1. Introduction

Automatic modulation classification (AMC) refers to the practical and accurate identification of modulated signals in the case of insufficient prior information [1]. When receiving over-the-air signals in a complicated environment, it is a priority to recognize the modulation types of signals before decoding the received signal. In both the civil and military areas, AMC plays an important role in complex wireless non-cooperative communication environments [2]. The methods employed to solve AMC problems can largely be divided into two categories: one is based on maximum likelihood theory and the other is feature based [3]. The maximum likelihood methods, which incorporate decision theory and hypothesis testing theory, achieve better performance; however, they consume a large amount of computational resources and require a great deal of prior knowledge [4]. By contrast, feature-based methods typically extract features in the pre-processing stage and apply some neural networks as optional classifiers to recognize diverse modulation types [5]. The extracted features can describe the intrinsic characteristics of the signals and reflect the differences between them. Feature-based methods are robust to noise and benefit from decent explainability. Moreover, the whole calculation process for feature-based methods is relatively more concise than that for the maximum likelihood methods. Accordingly, feature-based methods are commonly and effectively applied to solve AMC problems and are widely used in different environments [6].

Multiple amplitude-shift keying (MASK), multiple phase-shift keying (MPSK), and multiple quadrature amplitude keying (MQAM) are commonly used in communication systems for AMC. MASK often appears in scenarios with low carrier wave frequency, such as radio frequency (RF) applications and industrial networks. This modulation method is easy to realize and has low computational resource requirements. MPSK is a digital modulation method with high spectrum utilization and strong anti-interference properties for noise on amplitude, which is used in various communication systems such as optical satellite and cellular phone networks [7]. This method is adopted to increase signal bandwidth and reduce incorrect transmissions. In addition to MASK and MPSK, MQAM modulation has been widely used in satellite communications and microwave communications with modulation of amplitude and phase due to its higher spectral efficiency [8]. Nowadays, applying AMC techniques to MASK, MPSK, and MQAM signals is a very useful method of demodulating signals and obtaining the information carried therein across many common communication systems [9]. This technology can also judge the source site of the received signals, and manage the communication resources by prohibiting the unauthorized communication sender.

There are different kinds of features used for AMC, including wavelet transform, cyclic spectrum, and high-order cumulants (HOC) [10]. Wavelet transform is a multi-resolution method of time and frequency analysis which can decompose modulated signals on different scales. It can represent the details of various signals and recognize different types of digital modulation. However, wavelet transform requires too many attempts to search for the appropriate scale and translation factors. Therefore, it is not suitable to recognize modulated signals with little prior knowledge. In addition, cyclic spectrum features represent the correlation characteristics of digital signals and achieve decent anti-noise performance [11]. Cyclic spectrum features also have sparsity characteristics and highlight the position of peak points. Some researchers use different cyclic spectrum peak values to distinguish between various modulated digital signals [12]. Furthermore, cyclostationarity-based features are applied in [13] to recognize MQAM signals. However, these features are obtained via the Fourier series and include several variables; thus, their computational resource consumption is far higher when compared with HOC features. HOC can alleviate the side effects of the additive white Gaussian noise (AWGN) and effectively extract the characteristics of the original signals. The HOC features contain high-order information for use in distinguishing between different modulation types [14]. The different theoretical values of HOC make it feasible to recognize various modulated signals.

Common AMC classifiers mainly include support vector machine (SVM), subtractive clustering, and self-organizing map (SOM). Considering that the received signals are typically derived from non-cooperative systems, it is challenging to identify the modulation types of signals without adequate prior knowledge; this situation is exacerbated given that the received signals are always affected by various noise and interference. Because the extracted features usually have high dimensions, linear models such as SVM perform poorly when processing several dimensions of data and classifying various categories of samples [15]. Notably, however, the clustering method can overcome the side effects of the SVM approaches due to its ability to manage high-dimensional data in a nonlinear manner. Subtractive clustering can classify different types of modulated signals without setting the number of clusters ahead of time; however, the process of carefully adjusting the acceptance and rejection thresholds of the cluster density is complex and intricate [16]. Differently, SOM can learn the topological structure of the input samples and the distribution of the sample characteristics. It can also identify the intrinsic differences between signals and work without needing to set fine-tuned thresholds. Kaur et al. [17] utilize SOM to generate clusters from wavelet feature vectors. Zhou et al. [18] add an inherent partition mechanism to make the SOM neural network more suitable for recognizing modulation types and demodulating digital signals. Xu et al. [19] improve the SOM algorithm by adjusting the learning rate and adopting the neighborhood function. The improved model achieves better performance than both subtractive clustering and the SVM model. SOM is able to cluster modulated signals with clear boundaries and few computation resources.

SOM was first proposed by Kohonen [20] to imitate the human brain’s specific responses to external information [21]. As an unsupervised learning model, SOM is a type of network that can respond selectively to input data. In more detail, SOM is able to construct a topology between input features and output spaces. Meanwhile, it is also a self-learning network composed of fully connected neuron arrays without numerous training datasets and tags. SOM is widely applied in the fields of industry, finance, natural sciences, and linguistics [22]. Melin et al. [23] use SOM to analyze the ways in which similar countries fought the COVID-19 pandemic together and accordingly propose corresponding strategies. In [24], SOM dynamically switches between supervised and unsupervised learning during the training according to the availability of the class tags in the dataset. Hierarchical SOM [25] is an improved form of SOM that contains several SOM layers in a hierarchical structure. It can present an automated visualization scheme and produce better partitioning by using an effective knowledge representation method [26].

To present obvious clustering results and increase classification accuracies, the proposed hierarchical SOM model roughly clusters samples in the root layer and generates other neurons in the leaf layers to cluster samples of different modulation orders. HOC and amplitude moments are applied as effective features to describe the intrinsic differences between MASK, MPSK, and MQAM digital signals and distinguish these signals in the proposed two-layer SOM model. Moreover, a discrete transformation method based on modified activation functions is used to create obvious clusters in the leaf layer for MQAM signals. The proposed hierarchical SOM model is found to obtain higher classification accuracies and consume fewer computational resources when compared with some other common classifiers in the following parts.

The contributions of this paper are as follows:

- This paper proposes a method of generating leaf layers based on the root layer in a hierarchical SOM structure for AMC problems. This model can cluster the normalized features of different modulation types with diverse orders roughly in the root layer and finely in the leaf layers.
- This paper presents the discrete transformation based on modified activation functions for the features of MQAM samples. This kind of novel transformation method can create clear boundaries of clusters in the leaf layer and produce higher classification accuracies.
- This paper compares the hierarchical SOM model for automatic modulation classification with other common models. The proposed model can recognize more modulation types with higher classification accuracies and fewer computational resources.

The remainder of this paper is organized as follows. Section 2 introduces the selected features as the input of the SOM network. Section 3 provides the structure and learning procedures of the hierarchical SOM model. Section 4 discusses the discrete transformation based on modified activation functions for QAM features. The signal simulation method and experimental results are presented in Section 5, followed by a discussion and a conclusion.

## 2. Construction of Feature Space

At the receiving part, the down-converted and sampled signal can be expressed as follows:

(1) $$r\left(n\right)=ae^{j\left(2\pi f_{0}n+\theta_{0}\right)}s\left(n\right)+w\left(n\right)$$

where *a* is the attenuation factor, *n* is the time index of the baseband signal, $f_{0}$ is the signal frequency offset, $\theta_{0}$ is the phase offset, s(n) is the transmitted symbol coming from M types of modulation signals, and w(n) is the complex AWGN with constant power.

HOC features have different characteristics when compared to the signal spectrum and the wavelet transform. Several cumulants have recently been proposed as features in the literature for addressing the AMC problem [27]. Cumulant-based methods have been widely used to obtain superior classification accuracy, and almost all previous works of this kind have considered the calculation of different combinations of cumulant features that are feasible for classifiers. This process aims to extract the features that can obtain high resolution and strong robustness at a low signal-to-noise ratio (SNR) [28]. The following expressions are used to calculate HOC based on signal amplitude moments.

The p-order mixing moment of the random process r(t) is

(2) $$M_{pq}=E\left[r{\left(t\right)}^{p-q}r^{*}{\left(t\right)}^{q}\right]$$

where $r^{*}\left(t\right)$ is the conjugate complex value of r(t), while E[·] represents the mathematical expectation. The high-order cumulant of a zero-mean *k*-order stationary random process r(t) is defined as

(3) $$\begin{array}{cc} \; & C_{kr}\left(\tau_{1},\tau_{2},\cdots ,\tau_{k-1}\right)\\ = & \mathrm{Cum}\left(r\left(t\right),r\left(t+\tau_{1}\right),\cdots ,r\left(t+\tau_{k-1}\right)\right) \end{array}$$

where $\mathrm{Cum}\left(\cdot \right)$ refers to the high-order cumulant of r(t) and τ is the time delay of r(t). If r(t) is a Gaussian process, the value of mixing moments with more than two orders will be equal to zero. Therefore, the cumulants with three or more orders are extremely well suited to suppress Gaussian noise [14]. A Gaussian process is expressed in terms of HOC below.

(4) $$C_{kr}=\mathrm{Cum}\left(\tau_{1},\tau_{2},\cdots ,\tau_{k-1}\right)\equiv 0,k>2$$

As a result, these features are robust to interference from Gaussian noise. Moreover, the absolute values of these features are independent of initial phases, which means that they are robust to interference from different initial phases. The theoretical values of cumulants can be obtained by certain formulas. With reference to some related studies [29,30,31], we can determine that the $C_{40}$ and $C_{42}$ cumulants of MASK, MPSK, and MQAM are different from each other; thus, they can serve as effective features for classifiers. The formulas for the $C_{40}$ and $C_{42}$ cumulants of various orders are as follows.

(5) $$C_{40}=\mathrm{Cum}\left(x,x,x,x\right)=M_{40}-3M_{20}^{2}$$

(6) $$C_{42}=\mathrm{Cum}\left(x,x,x^{*},x^{*}\right)=M_{42}-2M_{21}^{2}-{\left|M_{20}\right|}^{2}$$

This paper constructs a feature space based on the HOC features outlined above to cluster feature samples. The first feature in this space is $|C_{42}|$. The theoretical $|C_{42}|$ values of MPSK signals are greater than those of MQAM signals, meaning that it is feasible to cluster MPSK or MQAM samples in the root layer. The second feature in the feature space is $|C_{40}|$. The PSK signals with various orders differ distinctly in terms of their $|C_{40}|$ values, meaning that PSK samples are able to be clustered in the leaf layer based on the $|C_{40}|$ feature.

However, the differences between 16QAM and 64QAM samples are difficult to distinguish. Accordingly, this paper applies an amplitude moment feature called $\mu_{42}$ to distinguish different orders of QAM samples. $\mu_{42}$, which is also called the kurtosis of instantaneous amplitude, can describe the degree of compactness for the received signals: more specifically, signals with bigger compact amplitude values often have larger $\mu_{42}$ values. The theoretical $\mu_{42}$ value of 16QAM is 2.3590, while that of 64QAM reaches 2.5025 [32]. The differences in the $\mu_{42}$ values of two kinds of QAM signals are accordingly obvious. The expressions of $\mu_{42}$ are defined below.

(7) $$\begin{array}{cc} \mu_{42}= & \frac{E\left[A_{c}^{4}\left(n\right)\right]}{E^{2}\left[A_{c}^{2}\left(n\right)\right]}\\ A_{c}\left(n\right)=\frac{A(n)}{m_{a}} & -1,m_{a}=\frac{1}{N}\sum_{n=1}^{N}A\left(n\right) \end{array}$$

In the above expressions, $A_{c}\left(n\right)$ is the central normalized instantaneous amplitude of the received signal r(n), A(n) is the instantaneous amplitude of the received signal r(n), and $m_{a}$ is the mean amplitude of one signal sample. $A_{c}$ can reflect the mean amplitude of the fluctuation signals without the side effects of direct current components.

The theoretical values of these features are in Table 1. Here, *E* is the energy value of the received signal; moreover, we use the $\mu_{42}$ of MQAM signals rather than MPSK signals to cluster samples in the leaf layer. These three features can be effectively used to distinguish between all specified modulation types. The number of samples generated for each modulation type is *S* and the total number of samples is 7S. The constructed feature space is a three-dimensional space containing several vectors. The feature of the *i*-th sample is expressed as $X_{i*}$ in the equation below.

(8) $$X_{i*}={\left[C_{42},C_{40},\mu_{42}\right]}_{i}\;i=1,2,\cdots ,7S$$

It should be noted here that the performance of the clustering model is based on the construction of features. To avoid interference from the energy of different modulated signals, it is necessary to apply a normalization method to the feature space. This paper selects the Min-Max scaling method to maintain the relative relationship of the original space. Every feature sample is scaled independently based on the same feature. The expression for the Min-Max normalization method is presented below.

(9) $${\hat{X}}_{ij}=\frac{X_{ij}-Min\left(X_{ij}\right)}{Max\left(X_{ij}\right)-Min\left(X_{ij}\right)}\;\left\{\begin{array}{c} j\;=\;1,i\in [1,7S]\\ j\;=\;2,i\in [3S\;+\;1,5S]\\ j\;=\;3,i\in [5S\;+\;1,7S] \end{array}\right.$$

where *i* indicates the index of the samples, while *j* refers to the *j*-th feature of the original space. $Max(X_{ij})$ is the maximum value of the *j*-th feature when the indexes are within the specified range and $Min(X_{ij})$ is the minimum value. This normalization method attempts to regularize samples so that every sample can be transformed into a unit norm for each feature, which is expressed as ${\hat{X}}_{ij}$. The values of ${\hat{X}}_{ij}$ range from zero to one. The feature samples are the input of the proposed model.

## 3. Structure and Algorithm of the Proposed Hierarchical SOM Model

SOM is an unsupervised learning system in which a group of connected neurons iteratively adapt to the distribution of input samples in the feature space. The remarkable characteristic of this model is that it maps similar input vectors to neighboring nodes in the output space. In essence, this mapping is a kind of reproduction of the original data, because it preserves the basic structure of the input space; it can learn the topological structure of the feature space consisting of the input samples in order to cluster similar samples near the mapping neuron in the output layer [33]. SOM is a vector-to-coding model that provides topological mapping to transform the input vectors into a topology with a specific pattern. Discrete mapping is carried out based on input feature samples with specified dimensions. This mapping is a self-adaptive process that progresses in a topological and orderly manner.

The proposed hierarchical SOM model is able to cluster numerous input vectors by finding a smaller number of prototypes to provide a good approximation of the original input space. The theoretical foundation of the hierarchical SOM model is rooted in vector quantization theory, the motivation for which is dimensionality control or data compression. Thus, the hierarchical SOM model can approximate the original space with different categories and use fewer computational resources in the process.

### 3.1. Hierarchical Structure of the SOM Model

**Figure 1:**
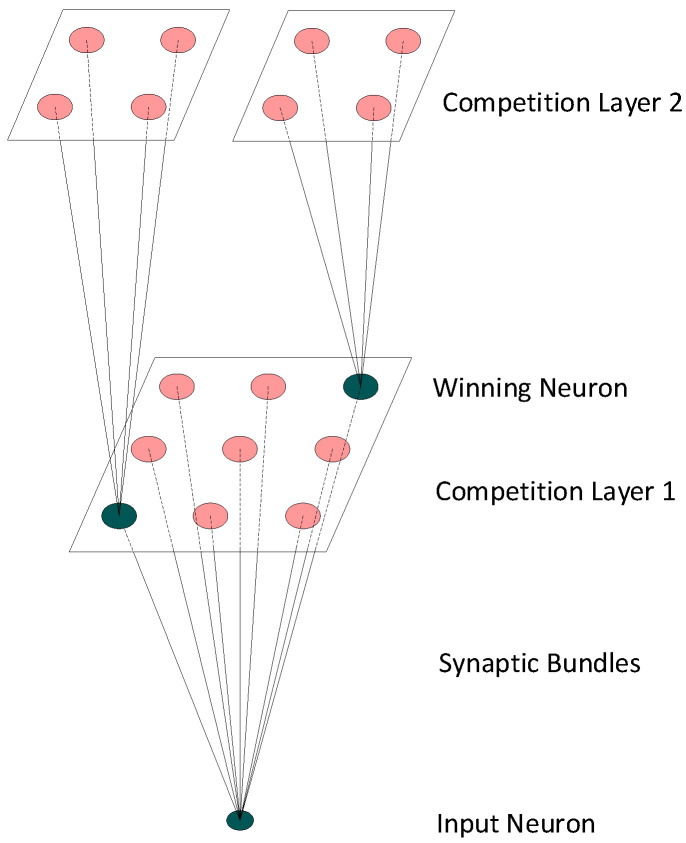
The structure of the hierarchical SOM model.

SOM simulates the connections among neurons in the human brain to achieve information processing according to biodynamic principles. Based on the conventional SOM structure with only one competition layer, the hierarchical SOM structure is proposed here to overcome the drawbacks of the former, which include high computational consumption and low precision. This hierarchical structure can cluster the input vectors more finely based on the output in the root layer. For each neuron in the leaf layer of the hierarchical SOM, the input data are the vectors connected with the winning neuron in the root competition layer [34]. Different features of the vectors are applied to cluster the samples in the leaf layer. These input data mapped to the same winning neuron usually have similar values or topological characteristics, enabling similar data to be clustered in the new layer. The structure of the hierarchical SOM with two layers is presented in Figure 1.

The neurons in the leaf competition layer connect with all winning neurons in the root competition layer. The flexible and layering hierarchical SOM can produce more detailed clustering results than the original SOM. The hierarchical SOM works in the data clustering phase to finely cluster input samples with their representative features and pattern. Each SOM layer uses one of three features to increase the classification efficiency [35]. The goal of hierarchical SOM is to transform a classification problem with large categories into multiple small problems with few categories to improve classification accuracy. This hierarchical model is based on the idea of the dividing and ruling technique. To cluster different categories in a hierarchical SOM structure, we cluster the large categories with the root SOM layer and small categories of different modulation orders with the leaf SOM layer.

A significant feature of a hierarchical SOM network is that it retains the topological structure of the input space so that the adjacent sample data in the input space will be mapped to adjacent neurons. We calculate the position of the winning node corresponding to each input sample; moreover, the neurons in the output plane will reflect the number of categories with clustering results. We can directly obtain the recognition result through the category tag of the highest frequency within the neurons of the output layer. Another valid choice would be to use the frequency of winning neurons to estimate the probability density of each category. When a new sample has a certain possibility of falling into a specific neuron on the output plane, we can determine the recognition result accordingly. The clustering results in the output layer are derived from the hierarchical mapping.

### 3.2. Algorithm of the Hierarchical SOM Model

The proposed hierarchical SOM neural network mapping method has five main processes: initialization for weight vectors, competitive learning for the winning neuron, cooperation based on topological neighborhood function, adjustment for weight vectors, and the generation of new layers. It gradually optimizes the network based on competition among neurons and maintains the topology of the input space through the neighborhood function. In a departure from the existing literature on classical SOM models [36], the hierarchical SOM model also adds a layering procedure to generate a new mapping layer for accurate classification. The algorithm of the hierarchical model is shown in Algorithm 1.
**Algorithm 1** Algorithm of the hierarchical SOM model**Require:** Input the feature of samples $x_{i}\;\left(i=1,2,\cdots ,M\right)$ **Ensure:** Output clustering results in the root and leaf layers  1: Initialize weight vectors $w_{j}\;\left(j=1,2,\cdots ,N\right)$ randomly  2: **for**
t=0 to T−1 **do**  3:    Find the winning neuron $j^{*}=\arg\min∥x_{i}-w_{j}∥$  4:    Define the topological neighborhood function near the winning neuron $N_{j^{*},k}=\exp\left(-\frac{d_{j^{*},k}^{2}}{2\sigma^{2}}\right)\;\left(k=1,2,...,P\right)$  5:    Adjust the weights of neurons in the topological neighborhood $w_{k}\left(t+1\right)=w_{k}\left(t\right)+\eta \left(t\right)\left(x_{i}-w_{j^{*}}\right)N_{j^{*},k}$  6:    **if** $qe=\frac{1}{M}\sum_{i=1}^{M}∥x_{i}-w_{j^{*}}∥<\tau$ **then**  7:      Generate the leaf layer and break  8:    **end if**  9:  **end for**  10: Obtain the clustering results in the root layer  11: Re-execute line 1 to line 5 for the leaf layers  12: Obtain the clustering results in the leaf layers

We first need to set up the size of the SOM layer and initialize weight vectors with small values. In normal cases, it is necessary to consider that the ideal distribution of the initial weights roughly approximates the distribution of the input sample. Random initialization uses random numbers in a small range to initialize the original weight vectors and is suitable in cases where little or no prior knowledge of the input data is available. This initialization method starts the weight vectors from an arbitrary initial state [22]. The advantage of this approach is that the output network nodes are similar to the topology of the input data at the beginning and converge to a fixed topology during training. In most datasets with nonlinear characteristics and an unbalanced distribution of values, random initiation performs better.

The competition process aims to find the winning neuron in the competition layer whose connected weight vector is the most similar to the input vector $x_{i}$(i=1,2,...,M), where *M* is the whole number. Moreover, the weight vectors are $w_{j}$(j=1,2,...,N), where *N* is the number of neurons in the output competition layer. The discriminant function of competition works by finding the minimum Euclidean distance between the input vector $x_{i}$ and the weight vector $w_{j}$ of each neuron in the competition layer [36], which is as follows.

(10) $$j^{*}=\underset{j}{\arg\min}∥x_{i}-w_{j}∥,i=1,2,\ldots ,M$$

where each input vector $x_{i}$ in an SOM neural network has its winning neuron, which is the winner of the competition among weight neurons. If several weight vectors fulfill the discriminant function, the vector with the smallest subscript is the winner. The weight neuron that satisfies the discriminance and subscript conditions is referred to as the winning neuron.

When a winning neuron is active, the nearest neighbor nodes tend to be more excited than more distant neighbor nodes. A topological neighborhood function $N_{j,k}\left(j=1,2,...,N;k=1,2,...,P\right)$ represents the influence area of the winning neuron *j* on the cooperative neuron *k* and the number of cooperative neurons is *P*. The winning neuron of the SOM neural network affects other neurons around it from near to far, and the effect weakens gradually from neural excitement to neural inhibition [37]. Therefore, the learning algorithm of the SOM neural network adjusts both the weight vector of the winning neuron and the weight vector of the surrounding neurons to varying degrees under the influence of the topological neighborhood function. The common neighborhood topology takes the form of a square, specifically four neurons in a square surrounding the winner neurons. $N_{j,k}$ is the topological neighborhood function centered on the winning neuron *j* and contains a group of cooperative neurons. One of these cooperative neurons is the neuron *k*. $d_{j,k}$ is the lateral distance between the winning neuron *j* and the excitatory neuron *k*. The topological neighborhood takes the maximum value when $d_{j,k}$ is both equal to zero and symmetrical about zero. The amplitude value of the neighborhood function $N_{j,k}$ decreases gradually with the increase of the lateral distance $d_{j,k}$. The expression of the common Gaussian topological neighborhood function is as below.

(11) $$N_{j^{*},k}=\exp\left(-\frac{d_{j^{*},k}^{2}}{2\sigma^{2}}\right)$$

where σ is the shape parameter that controls the width of the function peak. Specifically, it indicates the degree of participation of excitatory neurons near the winning neuron in the learning process. In practical use, the positive values of the Gaussian function can bring the weight vectors in the neighborhood closer to the winning neuron.

After determining the topological neighborhood, SOM performs an adjustment process through which the output nodes self-organize to establish a regular feature mapping. Not only will the winning neuron cause the weights to update, but its neighbors will also update their weights [38]. The adjustment process strengthens the response of the winning neuron to a subsequent similar input vector. This adaptive process provides accurate statistical quantification of the input vectors. The adjustment process will end if the number of iterations reaches the maximum value *T* for convergence. The updating formula of the weight vectors in the neighborhood area, including the weight vector of the winning neuron, for the *t*-th step is as follows.

(12) $$w_{k}\left(t+1\right)=w_{k}\left(t\right)+\eta \left(t\right)\left(x_{i}-w_{j^{*}}\right)N_{j^{*},k}$$

where η(t) is the learning rate and $N_{j,k}\left(t\right)$ is the topological neighborhood function for the *t*-th step. Setting the original learning rate as η(0), the iteration expression of η(t) is as below.

(13) $$\eta \left(t\right)=\eta \left(0\right)\frac{1}{1+2t/T},t=1,2,...,T$$

where *T* is the maximum number of iterations and η(t) decreases as *t* gradually increases. The decrease in the learning rate causes the SOM model to converge faster. Similarly, the shape parameter σ also becomes smaller with a larger number of iterations. The expression of the shape parameter for the *t*-th iteration, which is called σ(t), is shown below.

(14) $$\sigma \left(t\right)=\sigma \left(0\right)\frac{1}{1+2t/T},t=1,2,...,T$$

The SOM model applies the quantization error to generate the new layers. The quantization error is the average distance between every input sample $x_{i}$ and its best matching unit $w_{j^{*}}$. The new layer grows from the root SOM layer according to the quantization error in the original layer if the quantization error qe for the input samples in the competition layer fulfills the following network layering condition:

(15) $$qe=\frac{1}{M}\sum_{i=1}^{M}∥x_{i}-w_{j^{*}}∥<\tau ,t=1,2,\ldots ,T$$

where *M* is the number of input vectors, $j^{*}$ is the index of the best matching unit in the competition layer, ||.|| denotes the Euclidean distance between the input vector and the weight vector that connects the input vector and neuron in the competition layer, τ is the parameter that controls the generation of the leaf layer, and *T* is the maximum number of iterations. qe is able to measure the dispersion of all data mapped to a specific winning neuron and control the mapping procedure of the hierarchical SOM network. When a training iteration of the root layer ends, the algorithm will check whether all neurons on this layer meet the network layering condition. For any neuron *j* that fulfills this condition, a new layer is generated based on the root layer. For the neurons in the leaf layer of the hierarchical network, the input data are the sub-sequence of the original data that are mapped from the corresponding neurons in the root layer. Each layer in the model has a different size depending on the value distribution of the input features. When the new layer is generated from the root layer, the model returns to the initialization step for training with the original vectors like the root layer. If the number of iterations for neurons in the new layer eventually reaches the given iteration times, the proposed hierarchical SOM algorithm for clustering samples will terminate.

## 4. Discrete Transformation Based on Modified Activation Functions for QAM Features

As noted in Table 1, the differences between the theoretical values of 16QAM and 64QAM are almost imperceptible. To enhance the differentiation of the two QAM digital samples in the feature space, it is practical to apply a discrete transformation based on modified activation functions before clustering the specified features within the leaf layer. In fact, this kind of function makes the output vectors more discrete, meaning that the SOM network can distinctly cluster the output vectors with different orders of modulation. In this paper, the modified activation functions in the neural networks can complete the discrete transformation to map the original data into the specified interval ranges from zero to one. The discretized operation has no effects on the size of the SOM network and decreases the number of iterations required for convergence when training the leaf SOM layer. A number of activation functions, such as the arctangent function, can be employed to achieve discrete transformation. Moreover, the logistic function and hard-logistic function can also be modified to improve discrete transformation. We use scale (scale>0) to control the slope and shape of these functions. Through the application of these discrete transformation functions, the uniform input domain can be mapped to the discretized domain.

### 4.1. Modified Arctangent Function

The arctangent function can discretely map the original numerical values to a certain interval. The range of the arctangent function arctan(x) is between −π/2 and π/2, while the slope of this function at the zero point is 1. Hence, we need to scale the range of the original arctangent function and shift the center of the function to meet the mapping range requirements and set the slope at (0.5, 0.5). The modified arctangent function is as follows:

(16) $$\mathrm{mod}\text{-}arctan\left(x\right)=\frac{1}{\pi }arctan\left(0.25\pi *scale*\left(x-0.5\right)\right)+0.5$$

The modified arctangent function can suppress the input vectors when they are smaller than 0.5 and activate them when they are larger than 0.5. The value of scale can be set to adjust the slope of the modified arctangent function and obtain different discrete transformation effects; a larger value of scale results in a more pronounced discrete transformation. However, the range of output values is smaller than one and the effects of activation and inhibition are not prominent. As a result, it is necessary to modify the logistic function, which is commonly used in neural networks and has a wide output range and remarkable activation effects.

### 4.2. Modified Logistic Function

The common logistic function is defined as follows:

(17) $$\sigma \left(x\right)=\frac{1}{1+\exp(-x)}$$

This function can be considered an extrusion function that extrudes the input from a real field to a range between zero and one. When the input value is near zero, the logistic function is approximately linear. Input values are suppressed when they are sufficiently small. The smaller the input, the closer it is to zero; the larger the input, the closer it is to one. This behavior somewhat resembles that of biological neurons, which respond to some inputs with an output of one and suppress other inputs with an output of zero. Compared with the step activation function used by the perceptron, the logistic function is continuous and has better mathematical properties.

Due to the nature of the logistic function, the neuron equipped with the logistic activation function has the following two properties: first, its expression can be directly regarded as a probability distribution function, so that the neural network can be better combined with the statistical learning model; second, it can be regarded as a soft gate that controls the amount of information output from other neurons. To ensure that the input and output range is between zero and one and the slope of the function is adjustable at 0.5, we modify the logistic function as follows:

(18) $$\mathrm{mod}\text{-}\sigma \left(x\right)=\frac{1}{1+\exp(-scale*(x-0.5))}$$

where scale is the slope adjustment parameter used to adjust the slope of the function at zero. The large value of scale represents the very large slope of the function at 0.5.

### 4.3. Modified Hard-Logistic Function

The logistic and arctangent functions are both "s"-shaped functions with saturation characteristics, but the calculation cost is high, as these two functions are nonlinear in the saturation range at the left and right ends. Therefore, the logistic function can be approximated by piecewise functions. The derivative of the logistic function σ(x) is $\sigma^{\prime }\left(x\right)=\sigma \left(x\right)\left(1-\sigma \left(x\right)\right)$. The first-order Taylor expansion near zero of σ(x) is $g_{l}\left(x\right)$, which is approximated by $\sigma \left(0\right)+x\times \sigma^{\prime }\left(0\right)=0.25x+0.5$. It, therefore, follows that the function can be approximated by a piecewise hard-logistic function, which is defined by the equation below.

(19) $$\begin{array}{cc} \mathrm{hard}\text{-}\mathrm{logistic}\;(x) & =\left\{\begin{array}{cc} 1, & x\geq 2\\ g_{l}\left(x\right), & -2<x<2\\ 0, & x\leq -2 \end{array}\right.\\ \; & =\max\left(\min\left(g_{l}\left(x\right),1\right),0\right)\\ \; & =\max(\min(0.25x+0.5,1),0) \end{array}$$

The scale parameter also needs to be introduced into the hard-logistic function. To guarantee that the slope of the function is adjustable at 0.5 and the input and output range is between zero and one, we modify the hard-logistic function as follows:

(20) $$\begin{array}{cc} \; & \;\mathrm{mod}\text{-}\mathrm{hard}\text{-}\mathrm{logistic}\;(x)\\ \; & =\left\{\begin{array}{cc} 1, & x\geq x_{2}\\ g_{l}\left(scale*\left(x-0.5\right)\right), & x_{1}<x<x_{2}\\ 0, & x\leq x_{1} \end{array}\right.\\ \; & =\max\left(\min\left(g_{l}\left(scale*\left(x-0.5\right)\right),1\right),0\right)\\ \; & =\max(\min(0.25*scale*(x-0.5)+0.5,1),0) \end{array}$$

where $x_{1}$ is −2/scale+0.5 and $x_{2}$ is 2/scale+0.5. Here, $x_{1}$ is the left turning point and $x_{2}$ is the right turning point of the mod-hard-logistic function.

**Figure 2:**
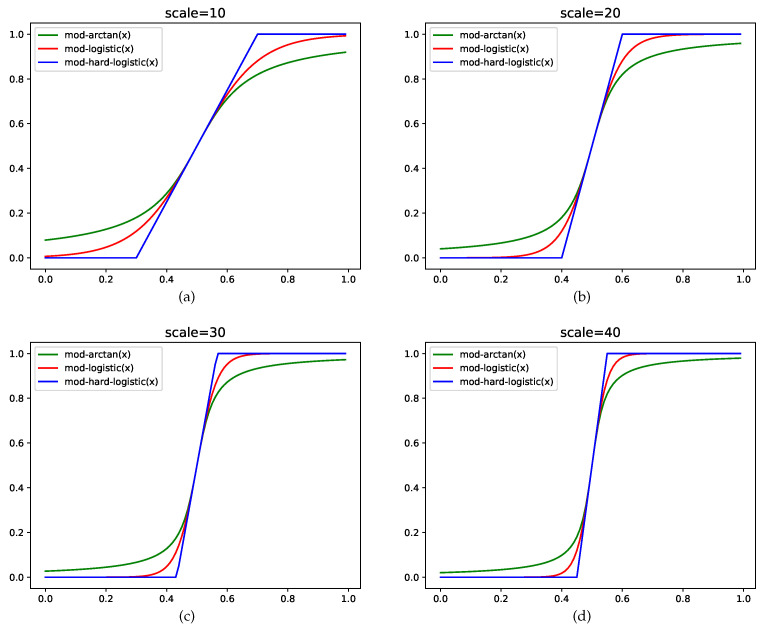
The modified functions with different values of scale.

With these discrete transformation functions, the unified input domain can be mapped to the binarized domain between zero and one. The slope at 0.5 of the mod-arctangent function is the same as that of mod-σ(x) and mod-hard-logistic(*x*). These three discrete transformation functions all have activation and inhibition effects. The function graphs of these functions with different scale values are in plotted Figure 2.

## 5. Simulation and Results

In this section, simulation and experimental results are presented to demonstrate the practicality and effectiveness of the proposed model on a deep learning platform with Python coding. The symbol rate and sampling rate of generated signals are considered, respectively. These signals exhibit some deviations, such as sampling rate deviation, carrier frequency offset, and the effects of AWGN. The modulation types of the generated signals include 4ASK, 8ASK, 2PSK, 4PSK, 8PSK, 16QAM, and 64QAM. These signals can simulate real-world signals with the relevant parameters. The number of samples for clustering is 2000 for each modulation type, such that the total number of samples is 14,000. The SNR value of the environment ranges from −5 dB to 10 dB. The signals are also affected by additive noise. The amplitude value of this kind of noise obeys a Gaussian distribution, while the power spectral density obeys a uniform distribution. The multiple interference factors convey the difficulty of distinguishing the specific modulation types. The different cumulants and amplitude moments in the constructed feature space can help to overcome these interference factors.

### 5.1. Clustering Results

**Figure 3:**
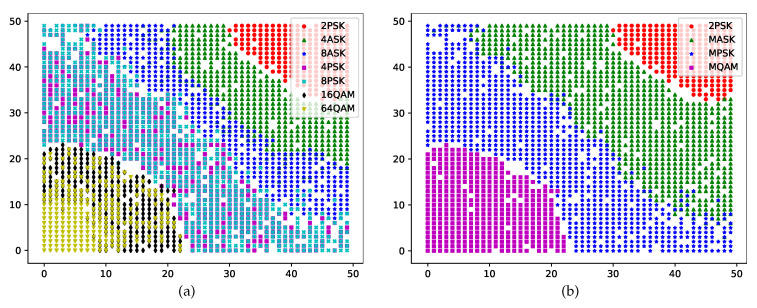
Clustering results in the root layer with different labels.

We first use the root layer to roughly cluster the 14,000 input samples. The length and width of this layer are both 50. From the description in Section 2, we know that the $|C_{42}|$ features of MASK, MPSK, and MQAM are different; thus, $|C_{42}|$ can be the classification feature of the root layer. The root layer maps the $|C_{42}|$ of the input samples and clusters them in different regions. The learning rate of modified activation functions η(0) is set to one. The clustering images in the root layer for the seven kinds of samples when the SNR is 10 dB are presented in Figure 3a. It is evident that the boundaries of 2PSK, 4ASK, and 8ASK are clear while the boundaries of 4PSK, 8PSK, 16QAM, and 64QAM are fuzzy. We, therefore, mark the latter group with four lags, namely 2PSK, MASK (including 4ASK and 8ASK), MPSK (including 4PSK and 8PSK), and MQAM (including 16QAM and 64QAM) in Figure 3b.

**Figure 4:**
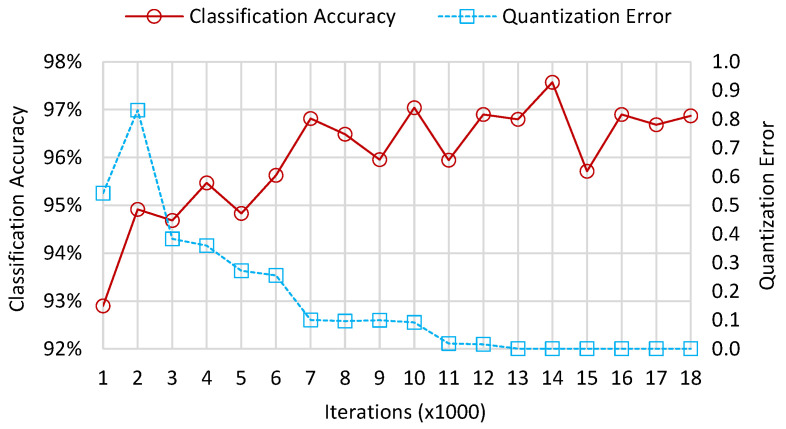
Classification accuracy and quantization error of the root layer.

The classification accuracy and the quantization error of the MASK, MPSK, and MQAM feature samples clustered in the root layer are presented in Figure 4. Here, classification accuracy is defined as the ratio between the number of correctly classified samples and the total number of feature samples. The quantization error is the average distance between each input sample and its best matching unit. As the number of iterations increases, the classification accuracy of the root layer climbs up before 7000 iterations, then fluctuates between 7000 and 18,000 iterations. The classification accuracy reaches its maximum of 97.6% when the number of iterations is 14,000. Moreover, the quantization error climbs from 0.542 to 0.832 when the number of iterations ranges from 1000 to 2000, then drops from 0.383 to 0.016 as the iterations increase from 3000 to 12,000. When the number of iterations exceeds 12,000, the quantization error converges to a fairly low value, less than the parameter τ=0.05, and remains at this level. At this range, the classification accuracy also converges to between 95.6% and 97.6% and does not increase further.

**Figure 5:**
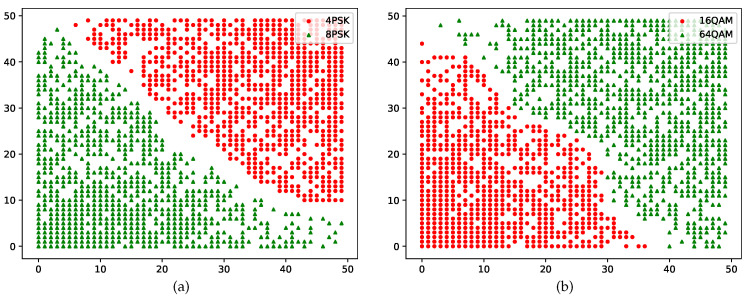
Clustering results of the leaf layers for MPSK and MQAM without discrete transformation.

To ensure that the MASK, MPSK, and MQAM samples cluster with clear boundaries, the hierarchical SOM network grows new leaf layers based on the root layer to distinguish between the candidate samples with different modulation orders. The leaf layer for MPSK clusters the samples with the second feature $|C_{40}|$, while that for MQAM clusters the samples with the third feature $\mu_{42}$. The output of the leaf layer is shown in Figure 5, including two categories of candidate MPSK (4PSK and 8PSK) and MQAM (16QAM and 64QAM) data; here, the length of the network is also 50. The clustering results with a clear boundary for MPSK samples in the leaf layers are presented in Figure 5a. The original clustering results for MQAM samples without modified activation functions in the leaf layers are presented in Figure 5b.

**Figure 6:**
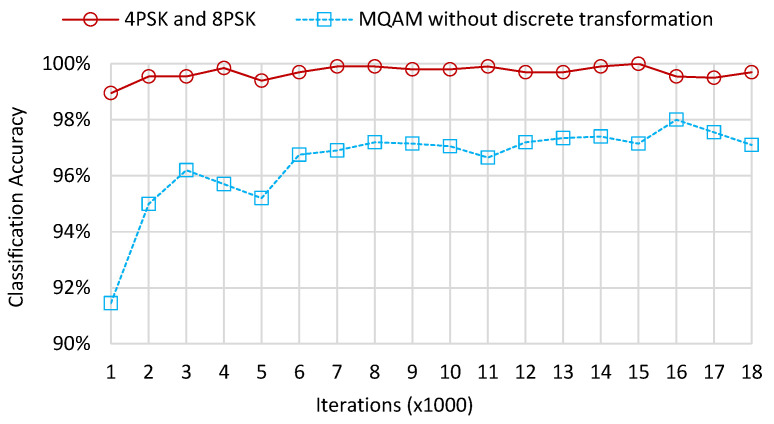
Classification accuracy of the leaf layers for MPSK and MQAM without discrete transformation.

However, the 16QAM and 64QAM samples cluster closely, which introduces bad clustering effects. Consequently, we apply a discrete transformation with modified activation functions to make the input vectors discretize and cluster more explicitly. The numerical size relationship between two kinds of QAM samples does not change due to the monotonicity of discrete transformation. Figure 6 illustrates the classification accuracy of the leaf layers without discrete transformation. The accuracy for 4PSK and 8PSK reaches 99.5% when the number of iterations is 1000 and remains high as iterations increase. Moreover, the accuracy for 16QAM and 64QAM is 94.1% at the beginning of iterations and increases to its maximum value of 97.9% when the number of iterations reaches 16,000.

**Figure 7:**
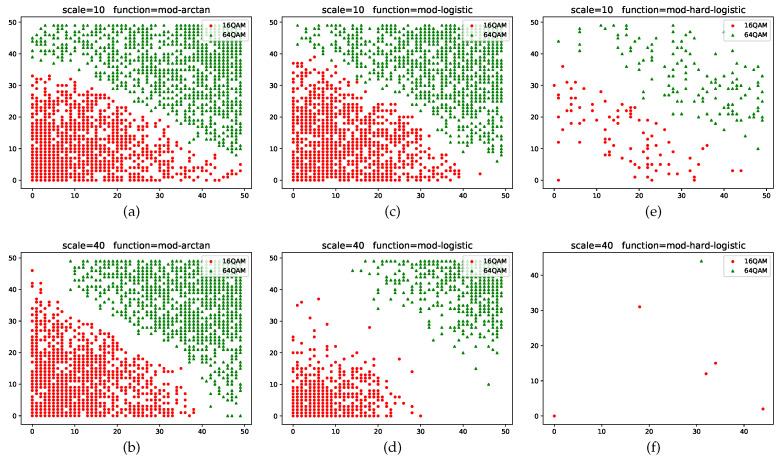
Clustering results of the leaf layer for MQAM with discrete transformation and different scales.

**Figure 8:**
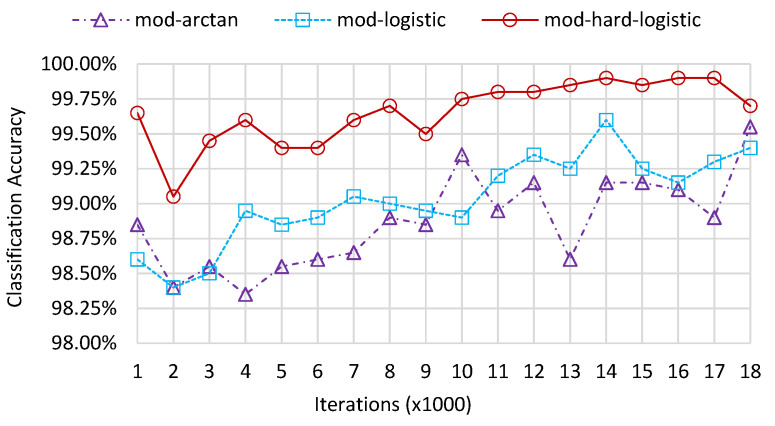
Classification accuracy of the leaf layer for MQAM with discrete transformation.

The clustering results of QAM samples with different orders through various modified activation functions with two distinct values of scale are presented in Figure 7. From the six contrasting images, we can observe that as the value of scale increases, the boundaries between two clusters become clear and the winning neurons in different regions tend to be more discretized. The clustering results of the mod-arctangent function are worse than those of the mod-logistic function, while the mod-hard-logistic function can be seen to have the best clustering effect, as the edges of the two clusters are far away from each other. This phenomenon results from the binarization characteristics of different activation functions near the minimum and maximum points. Most of the output data is clustered in such a way as to make the clustering results obvious. The classification accuracy of these three activation functions for discrete transformation is shown in Figure 8 when scale is 40. The accuracies with different numbers of iterations all exceed 98%. In general, the accuracy of clustering results obtained using the mod-arctangent function is lower than that obtained through the mod-logistic function, while the mod-hard-logistic function has the highest accuracy.

**Figure 9:**
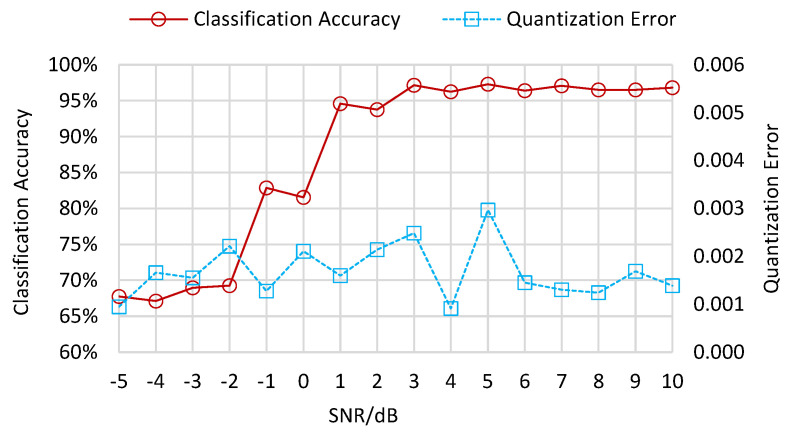
Classification accuracy and quantization error of the root layer under different SNRs.

**Figure 10:**
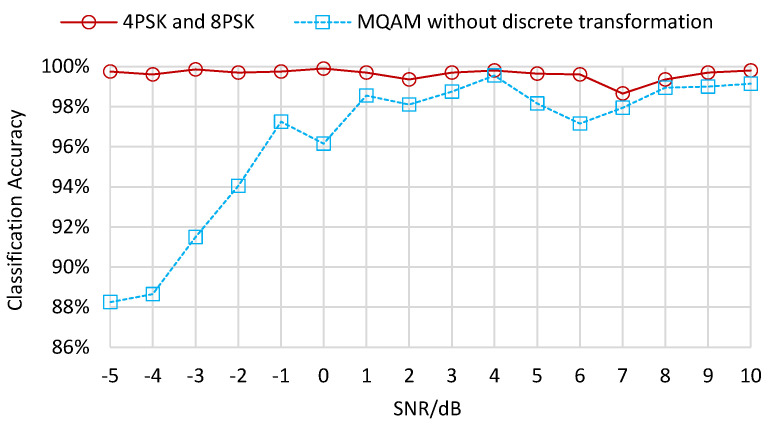
Classification accuracy of the leaf layers for MPSK and MQAM without discrete transformation under different SNRs.

**Figure 11:**
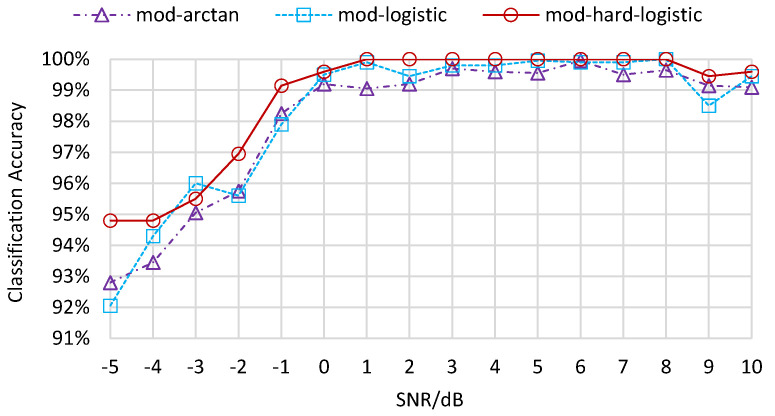
Classification accuracy of the leaf layer for MQAM with discrete transformation under different SNRs.

The experimental results above are under the SNR condition of 10 dB. As the SNR value ranges from −5 dB to 10 dB, the classification accuracies in the root layer present a trend from low value to high value, as shown in Figure 9. When the SNR is lower than −1 dB, the classification accuracies are smaller than 70%. When the SNR is between −1 dB and 1 dB, the classification accuracies increase unsteadily to 94.6%. Subsequently, the classification accuracies rise smoothly from 93.8% to 97.3% when the SNR ranges from 2 dB to 10 dB. As for the leaf layer results in Figure 10, the classification accuracies for MPSK samples remain high from −5 dB to 10 dB. The classification accuracies for MQAM samples without discrete transformation increase from 88.3% to 97.3% when the SNR ranges between −5 dB and −1 dB, then fluctuate between 96.2% and 99.6% when the SNR increases towards 10 dB. The classification accuracies for MQAM samples with three discrete transformations increase from −5 dB to 1 dB, then fluctuate between 2 dB and 10 dB, as shown in Figure 11. The accuracies obtained with the mod-hard-logistic function usually exceed those obtained using the mod-logistic function, followed by those obtained with the mod-arctangent function.

Compared with previous research into automatic modulation classification on four modulation categories, including 2PSK, 4PSK, 8PSK, and 16QAM, and using the methods of one sample 2D K-S classifier with parameter estimated by ECM (K-S classifier), a combination of genetic programming and k-nearest neighbor (GP-KNN classifier), clustering analysis of constellation signature (CACS classifier) [39], and maximum likelihood classifier with parameters estimated by SQUAREM-PC (ML classifier), our proposed hierarchical SOM model (H-SOM) can deal with more modulation types and obtain higher accuracy under the same SNR environment, as shown in Table 2. The methods in prior works extract spectral and statistical features, which can enhance the differences between the samples of interest. In our method, however, the hierarchical SOM model selects only statistical features, including high-order cumulants $C_{42}$ and amplitude moments $\mu_{42}$, based on recent research. $C_{42}$ can suppress the side-effects of additive Gaussian noise and demonstrates robustness when classifying MASK and MPSK signals. $\mu_{42}$ reflects the level of compactness for the received signals and works as an indicator to facilitate the classification of 16QAM and 64QAM signals. H-SOM is sufficiently sensitive to present the differences in input data in the output clustering layer.

### 5.2. Comparison of Computational Requirements

In practical applications, computational requirements play a crucial role when applying the proposed model for the inference stage after training. The inference stage is based on the existing parameters in the training stage. When training a sufficiently large amount of data to obtain a trustworthy model, it is necessary to consider the consumption of computational resources in order to reduce time costs and accelerate the inference stage. For some time-first scenarios, such as embedded applications and quick response systems, it is favorable to select the models with lower computational requirements to obtain better performance and reduce power consumption. When calculating the computational requirements, the mathematical operations applied to complete the classification are taken as the measure of the computational requirements for each classifier. We further make some assumptions to calculate the number of various operations as follows: (1) the total number of modulation types for classification is $T_{m}$; (2) the number of samples in the *i*-th modulation candidate is $M_{i}$; (3) the signal sample length is *N*; (4) the number of weights in the model is *W*; (5) the number of proposed neurons in the *i*-th layer of hierarchical SOM is $P_{i}$. The overall computational requirements of the maximum likelihood classifier (ML) and stacked auto-encoder-based deep neural network (SAE-DNN) classifiers are presented in [40], while the computational requirements of the inference stage of hierarchical SOM are simply related to the addition operations in the competition procedure, which are presented in Table 3. There are no extra computational resources required for the samples of 2PSK and MASK in the leaf layer.

We can observe that classifiers based on the ML theorem and SAE-DNN require multiplications and exponential operations. As the resource consumption of addition operations is far lower than that for multiplications and exponential operations and the number of proposed neurons is quite smaller than the models for comparison, we can make a conclusion that the computational requirements, including additions, multiplications, exponents, and logarithms, of the hierarchical SOM model are smaller than those of the other two models.

## 6. Discussion

The hierarchical SOM model can present the differences between groups of data and cluster similar features on a two-dimensional plane. The main advantage of using a hierarchical SOM is the clear visualization of clustering results. The mapping of feature samples in grid form makes it easy to present the similarities and clustering results of the data. SOM processes all data in the feature space to generate independent clusters. The nonlinear classification ability of SOM makes samples cluster in certain regions through the application of self-learning procedures. SOM can handle several classification problems by providing a functional, interactive, and intelligible outline of the data. The hierarchical SOM can cluster distinct samples roughly in the root layer, then cluster samples with subtle differences in the leaf layer. This model can cluster MQAM and MPSK samples based on the HOC and amplitude moment features of the input digital signals. The hierarchical SOM has the advantages of higher classification accuracy and lower computational resource consumption. Moreover, the clustering model is explainable, which makes the clustering results trustworthy according to the theoretical values of selected features. The samples with low feature values gather in the lower left corner of the output layer, while those with high values gather in the upper right corner of the output layer. The boundary line of clustering usually runs from the upper left to the lower right. We can find that the sums of horizontal and vertical coordinates are positively correlated with the feature values of samples.

One disadvantage of a hierarchical SOM is that it requires a sufficient number of iterations to generate clusters and obtain high accuracy. The weight vectors need to iterate more than 10,000 times to distinguish samples with high accuracy. The lack of adequate iterations for weight vectors will result in fuzzy boundaries of clusters and fluctuating classification accuracy. Moreover, it is also uncertain which iteration produces the highest accuracy, making it necessary to try enough iterations to choose the most eligible one in practical applications. Another disadvantage of hierarchical SOM is that the clustering results depend on the value distribution of the original dataset. Similar samples belonging to different categories may cause some samples to cluster in adjacent regions or even mix up in the same region. This phenomenon will unavoidably decrease the classification accuracy of the hierarchical SOM model. While the discrete transformation proposed in this paper can reduce the negative impacts of similar samples in different categories, it cannot eliminate this phenomenon completely. It is, therefore, practical to remove abnormal samples before using the hierarchical SOM model to get better clustering results in the output layer before the normalization of the features.

## 7. Conclusions

The hierarchical SOM model proposed in this paper applies stacked neuron layers to cluster MASK, MPSK, and MQAM digital samples based on the normalized HOC and amplitude moment features without the need to set thresholds based on these features. These features are robust to noise and show the differences between different modulation types. Unlike conventional one-layer SOM models, the hierarchical SOM model can generate leaf layers in the procedure of layering according to the values of quantization errors. This model can roughly cluster the MASK, MPSK, and MQAM samples in the root layer and then finely cluster the MPSK and MQAM samples in the leaf layers. In addition, this model can improve the visualization and classification results of clustering MQAM samples based on discrete transformation in the leaf layer. The classification accuracies of hierarchical SOM are higher than those of the four existing clustering methods selected for comparison. While hierarchical SOM is suitable for fine classification of some similar data, the model training parameters need much professional experience; this model requires a sufficient number of iterations in order for the clustering procedures to obtain high accuracy. In future work, it would be feasible to consider the application of this hierarchical SOM model in different clustering problems focusing on more categories of modulated signals or other types of similar data.

## Figures and Tables

**Table 1:** Index range and theoretical values of features of different modulation types.

Types	Index Range	$|C_{42}|$	$|C_{40}|$	$\mu_{42}$
4ASK	[1, *S*]	1.36*E*${}^{2}$	-	-
8ASK	[S+1, 2*S*]	1.24*E*${}^{2}$	-	-
2PSK	[2*S*+1, 3*S*]	2*E*${}^{2}$	-	-
4PSK	[3*S*+1, 4*S*]	*E* ${}^{2}$	*E* ${}^{2}$	-
8PSK	[4*S*+1, 5*S*]	*E* ${}^{2}$	0	-
16QAM	[5*S*+1, 6*S*]	0.68*E*${}^{2}$	-	2.3590
64QAM	[6*S*+1, 7*S*]	0.62*E*${}^{2}$	-	2.5025

**Table 2:** The accuracy results among different models.

Model Name	Modulation Types	SNR/dB	Accuracy/%
K-S classifier	2PSK, 4PSK, 8PSK, 16QAM	10	69.8
GP-KNN classifier	2PSK, 4PSK, 8PSK, 16QAM	10	70.9
CACS classifier	2PSK, 4PSK, 8PSK, 16QAM	10	85.2
ML classifier	2PSK, 4PSK, 8PSK, 16QAM	10	89.3
H-SOM classifier	MASK, MPSK, MQAM (7 types)	10	96.6

**Table 3:** Computational requirements of different classifiers.

Classifiers	Additions	Multiplications	Exponents	Logarithms
ML	$6NT_{m}\sum_{i=1}^{T_{m}}M_{i}$	$5NT_{m}\sum_{i=1}^{T_{m}}M_{i}$	$NT_{m}\sum_{i=1}^{T_{m}}M_{i}$	$NT_{m}$
SAE-DNN	$NWT_{m}\sum_{i=1}^{T_{m}}M_{i}$	$NWT_{m}\sum_{i=1}^{T_{m}}M_{i}$	$NT_{m}\sum_{i=1}^{T_{m}}M_{i}$	0
H-SOM	$P_{1}\sum_{i=1}^{T_{m}}M_{i}+P_{2}\sum_{i=2}^{T_{m}}M_{i}$	0	0	0
